# Dynamics of auditory cortical activity during behavioural engagement and auditory perception

**DOI:** 10.1038/ncomms14412

**Published:** 2017-02-08

**Authors:** Ioana Carcea, Michele N. Insanally, Robert C. Froemke

**Affiliations:** 1 Departments of Otolaryngology, Neuroscience and Physiology, Skirball Institute for Biomolecular Medicine, New York University School of Medicine, 540 First Avenue, New York, New York 10016, USA; 2 Center for Neural Science, New York University, New York, New York 10003, USA

## Abstract

Behavioural engagement can enhance sensory perception. However, the neuronal mechanisms by which behavioural states affect stimulus perception remain poorly understood. Here we record from single units in auditory cortex of rats performing a self-initiated go/no-go auditory task. Self-initiation transforms cortical tuning curves and bidirectionally modulates stimulus-evoked activity patterns and improves auditory detection and recognition. Trial self-initiation decreases the rate of spontaneous activity in the majority of recorded cells. Optogenetic disruption of cortical activity before and during tone presentation shows that these changes in evoked and spontaneous activity are important for sound perception. Thus, behavioural engagement can prepare cortical circuits for sensory processing by dynamically changing sound representation and by controlling the pattern of spontaneous activity.

## Abstract (web summary)

Sensory perception is enhanced with behavioural engagement. Here the authors show that when rats initiate stimulus delivery in an auditory recognition task, activity of auditory cortex neurons is modulated and optogenetic disruption of this activity affects performance.

Changes in brain state can control perceptual abilities by modulating the detection of sensory input in a background of ongoing activity and the recognition of behaviourally relevant inputs over less relevant or distracting inputs^1^,^2^. Neurophysiologically, many aspects of cortical activity and representations can be modulated to affect sensory processing including the structure of neuronal receptive fields^3^,^4^,^5^, population dynamics^6^,^7^,^8^, spike rates or spike timing during periods of stimulus presentation^9^,^10^,^11^,^12^, or patterns of spontaneous activity^13^,^14^,^15^,^16^,^17^,^18^,^19^.

Neurons in the auditory cortex fire in response to acoustic stimuli in a manner that depends on sound frequency and amplitude^3^,^4^,^20^. In the adult auditory cortex, sound representations are thought to generally be quite stable^21^,^22^. However, transient changes in cortical auditory receptive fields have been reported following various behavioural tasks and following biochemical manipulations. During learning and activation of several neuromodulatory systems changes in the gain and shape of synaptic and spiking cortical receptive fields were observed at the single cell level and at the population level^4^,^23^,^24^,^25^,^26^,^27^,^28^,^29^. These changes in cortical sound representations develop over minutes to hours, can last from hours to weeks and can improve the detection and the recognition of sounds^4^,^27^,^29^. More rapid changes in the activity of cortical neurons have been observed during movement, when spontaneous and evoked activity in the auditory cortex were suppressed by top–down projections from motor areas^30^,^31^. Similarly, behavioural task engagement and intermediate arousal states have been shown to induce generalized spiking suppression and membrane hyperpolarization engagement^32^,^33^,^34^,^35^,^36^ but the causal relationship between this type of modulation and perception is not clear. Behavioural engagement can also increase the activity of a restricted number of neurons^32^,^37^, can modulate spontaneous activity^14^ and can decrease noise correlations in the auditory cortex^38^. How the mixed modulations of responses in the auditory cortex by behavioural engagement contribute to performance remains unclear.

Depending on the demands of behavioural tasks, there are different modes and levels of engagement that can result in different performance. How do different forms of engagement modulate activity in the auditory cortex to impact perceptual detection and recognition? Here we examine this question using two different variants of a frequency recognition task, while monitoring neural activity in rat auditory cortex. We find that voluntarily initiating behavioural tasks modulates spontaneous and evoked neuronal activity in the auditory cortex to improve sound detection and recognition.

## Results

### Behavioural engagement improves auditory perception

**Figure 1:**
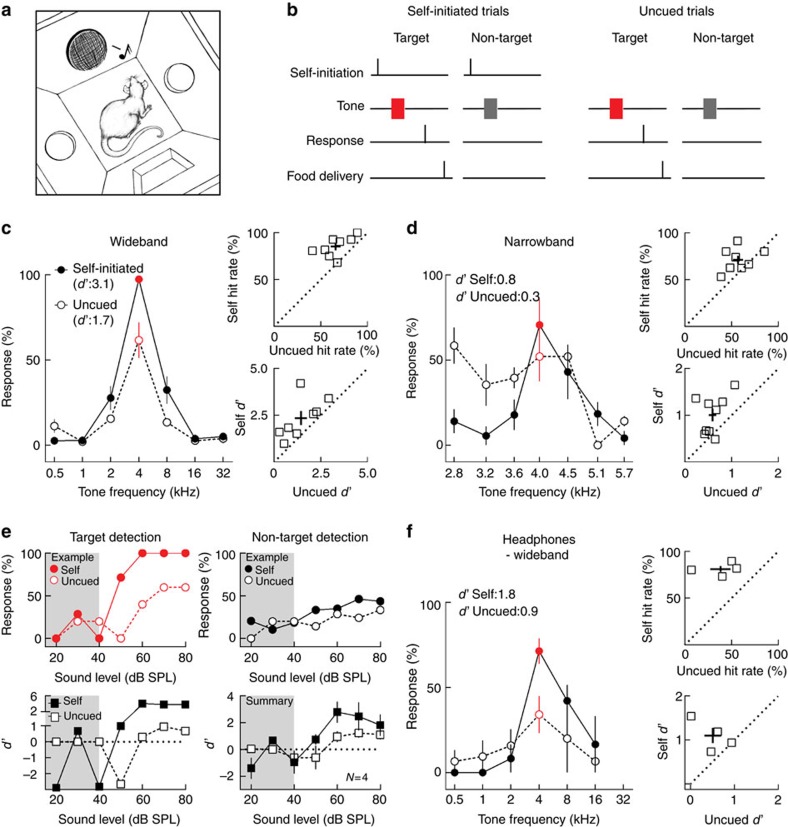
Self-initiated and uncued auditory target recognition. (**a**) Schematic of the operant conditioning chamber with two nose ports (one for self-initiation and one for target response), one speaker and one food dispenser. (**b**) Schematic of the go/no-go auditory behavioural task. Target (red) and non-target (grey) tones were 100 ms in duration, distributed one octave apart between 0.5 and 32 kHz, and delivered in a random order at 70 dB SPL. For the uncued trials, the animals did not self-initiate; instead, the trials were programmed to start at pseudo-random inter-trial intervals between 6 and 10 s. (**c**) Performance on the wideband stimulus set. Left, individual performance of one animal over three consecutive sessions of self-initiated trials (filled circles, solid line) or uncued trials (open circles, dashed line). In red, target tone (4 kHz); other tones were unrewarded non-targets. Top right, summary of hit rates for all animals. Hit rate was higher during self-initiation than uncued trials. Each square represents one animal. Bottom right, summary of *d*′ values for all animals. Stimulus recognition was higher during self-initiation than uncued trials. Error bars indicate mean and s.e.m. in both dimensions. (**d**) Performance on the narrowband stimulus set. Left, example individual performance when the target and non-target stimuli were at smaller perceptual distances from each other. Red, target tone (4 kHz). Filled circles and solid line, self-initiated trials. Empty circles and dashed line, uncued trials. Top right: summary plots showing hit rates for all rats during ‘Self' and ‘Uncued' trials. Bottom right: summary plots showing *d*′ values for all rats. (**e**) Performance on the detection task. Top left: example hit rates to the target frequency at different sound levels. Filled circles and solid line: self-initiated trials. Empty circles and dashed line: uncued trials. Shaded area represents responses to tones played below the background noise level (30–40 dB). Top right: the false alarm rate remained relatively low at all sound levels. Bottom: *d*′ values calculated for each tone level. Right: summary data showing *d*′ values for four rats. (**f**) behavioural performance when the stimuli were delivered via headphones. Left: example individual performance when the stimuli were presented via headphones. Right: summary plots showing performance for all rats with headphones during self-initiated and uncued trials. Error bars are s.e.m.

We used a behavioural training paradigm to control the mode and level of behavioural engagement in a total of 25 rats. Eight of these animals were first trained to nosepoke for food reward following a target tone and to withhold from nosepoking after non-target tones (Fig. 1a,b). We tested animal performance on consecutive blocks of two variants (‘self-initiate *d*′ and ‘uncued') of this frequency recognition go/no-go task. In the ‘self-initiate *d*′ variant, rats voluntarily engaged in the task by self-initiating the trials, with tones occurring at 0.5, 1 or 1.5 s after self-initiation (Fig. 1b and Supplementary Movie 1). In the ‘uncued' variant, trials were externally triggered by tone presentation at pseudo-random time intervals between 6 and 10 s (Fig. 1b and Supplementary Movie 2). Except for the mode of initiation, the rest of the behavioural task was identical between the self-initiated and uncued conditions. All stimuli were 0.5–32 kHz pure tones at one octave intervals, presented at 70 dB sound pressure level (SPL) and 100 ms in duration. The target frequency was either 4 or 16 kHz and other frequencies were non-targets.

As expected, animals reliably responded to the target tone and rarely responded to non-target tones during self-initiated trials compared with uncued trials (Fig. 1c left; responses to 4 kHz target increased from 61.6±10.4% during uncued trials to 97.3±1.5% during self-initiated trials in this animal, *n*=3 behavioural sessions, *P*=0.02, Student's unpaired two-tailed *t*-test; false alarm rate did not change significantly: 8.1±1.8% during uncued trials and 12.3±3.0% during self-initiated trials, *P*=0.3; *d*′ increased from 1.7 during uncued trials to 3.1 during self-initiated trials). This led to higher hit rates and improved the *d*′ discriminability index when the non-target frequencies were 1+ octaves from the target tone on a less-challenging ‘wideband' version of the stimulus set (Fig. 1c, right; hit rates during self-initiated trials: 85.3±3.7% and during uncued trials: 66.0±5.4%, *N*=8 rats, *P*=0.003, Student's paired two-tailed *t*-test; *d*′ during self-initiated trials: 2.4±0.4 and during uncued trials: 1.4±0.3, *N*=8 rats, *P*=0.02, Student's paired two-tailed *t*-test). Similar enhancements were observed when the target and non-target tones were spectrally closer together on a more-challenging ‘narrowband' version (Fig. 1d; left, example animal, responses to 4 kHz target did not change significantly from 52.1±14.5% during uncued trials to 70.6±15.1% during self-initiated trials, *n*=3 sessions, *P*=0.4, Student's unpaired two-tailed *t*-test, but false alarm rate decreased from 33.3±0.9% during uncued trials to 17.2±2.5% during self-initiated trials, *P*=0.002; *d*′ increased from 0.3 during uncued to 0.8 during self-initiated trials; right, summary, hit rates during self-initiated trials: 71.4±4.3% and uncued trials: 57.0±5.2%, *N*=8 rats, *P*=0.03, Student's paired two-tailed *t*-test; *d*′ during self-initiated trials: 1.0±0.1 and during uncued trials: 0.6±0.1, *P*=0.01).

To determine whether self-initiation could also improve detection abilities, we varied the amplitude of target and non-target tones between 20 and 80 dB SPL, against the background noise level in the behaviour box (30–40 dB SPL). During self-initiated trials, animals detected the target tones at lower amplitudes than during uncued trials (Fig. 1e; top left panel, example animal: hit rates at 50–60 dB SPL increased from 20.0±20.0% during uncued trials to 85.7±14.3% during self-initiated trials, *n*=29 trials, *P*=0.003, two-tailed Fisher's exact test). False alarms remained low at all tone amplitudes, indicating that rats correctly recognized the target from non-target tones even at low sound levels during both self-initiated and uncued trials (Fig. 1e; top right panel, false alarm rates at 50–60 dB SPL for uncued trials: 21.4±7.1% and self-initiated trials: 34.2±0.8%, *n*=133 trials, *P*=0.05, two-tailed Fisher's exact test; bottom left panel, *d*′ values for example animal for 50–60 dB SPL increased from −1.2±1.8 during uncued to 2.6±1.9 during self-initiated trials; bottom right panel, summary *d*′ values calculated for 50–60 dB SPL increased from 0.2±0.6 during uncued trials to 1.8±0.5 during self-initiated trials, *N*=4 rats, *P*=0.02, Student's paired two-tailed *t*-test).

Although the overall structure of the task and the significance of tones were the same between self-initiated and uncued trials, there could be differences in performance between these two versions related to the position of the rat in the behaviour box at tone onset, or the movement of the rat during the trial and the duration of inter-tone intervals. Therefore, we next controlled for the possible contribution of these parameters to the improved performance during self-initiated trials.

First, we quantified differences in the duration of inter-tone intervals and tone presentation rate between self-initiated and uncued trials; we found no significant differences between the two task variants (Supplementary Fig. 1). This means that during behavioural testing, the total number of trials was similar for self-initiated and uncued sessions.

Next, to better control the sound level irrespective of the position of the animal relative to the speaker, we bilaterally implanted small speakers in the ear canals of four rats (Supplementary Movie 3). Rats with these implanted headphones also had improved performance during self-initiated trials (Fig. 1f; left panel, example performance, hit rate to 4 kHz target increased from 34.2±10.9% during uncued trials to 71.4±7.5% during self-initiated trials, *n*=4 sessions, *P*=0.03, Student's unpaired two-tailed *t*-test; false alarm rates did not change significantly for this animal from 10.4±7.0 during uncued trials to 11.2±1.9 during self-initiated trials, *P*=0.9; *d*′ values increased from 0.9 during uncued to 1.8 during self-initiated trials; top right panel, summary data, hit rates increased from 38.2±10.8% during uncued trials to 81.1±3.3% during self-initiated trials, *N*=4 rats, *P*=0.02, Student's paired two-tailed *t*-test; bottom right panel, *d*′ increased from 0.8±0.3 during uncued to 1.7±0.2 during self-initiated trials, *P*=0.03). Incidentally, it appeared that performance using headphones was lower compared to stimuli presented through the free-field speaker. It is unclear why this might be, however we speculate that animals may have difficulty adapting to the change in ear pressure with chronic headphones in place. Nonetheless, there was a significant enhancement in behaviour on the self-initiated trials versus uncued trials, indicating that the neural mechanisms engaged by self-initiation remain intact in headphone-implanted animals.

To control for the movement of animals in the behavioural box, we tracked the *x*–*y* position in real time during both self-initiated and uncued tasks. As expected, there was more movement before some self-initiated trials (generally following hits or false alarms, when the animal had been moving from the nosepoke port or the food tray). As such, this usually occurred in a period of 2 s before trial initiation, which we refer to as ‘Interval A' for the self-initiated trials (Supplementary Fig. 2a–c, Interval A *x*-motion during self-initiated trials: 10.8±0.7 cm, Interval A *y*-motion during self-initiated trials: 4.4±0.3 cm, *n*=91 trials). However, during the following interval between trial self-initiation and tone onset 0.5–1.5 s later (‘Interval B'), animals maintained a relatively fixed position with minimal movement (Supplementary Fig. 2a,b, Interval B *x*-motion during self-initiated trials: 4.4±0.5 cm, Interval B *y*-motion during self-initiated trials: 4.0±0.4 cm, *n*=91 trials, one-way analysis of variance (ANOVA) with Tukey's multiple comparison test). Throughout the uncued trials, we defined Interval A as the interval between 3 and 1 s before tone onset and Interval B as the one second preceding tone onset. The animals had little movement in either Interval in both coordinates (Supplementary Fig. 2a–c Interval A *x*-motion in uncued trials: 2.7±0.5 cm; Interval B *x*-motion: 2.6±0.5 cm; Interval A *y*-motion in uncued trials: 4.4±0.4 cm; Interval B *y*-motion in uncued trials: 3.4±0.3 cm, *n*=78 trials, one-way ANOVA with Tukey's multiple comparison test). Differences in movement during self-initiated trials compared to uncued trials did not explain the improved behavioural performance, as the *x*- and *y*-motion during correct trials was similar to the motion during error self-initiated trials (Supplementary Fig. 2c,d, *x*-motion during correct trials: 3.4±0.8 cm and during error trials: 4.0±0.6 cm, *N*=4 rats, *P*=0.6, Wilcoxon matched-pairs two-tailed signed-rank test; *y*-motion during correct trials: 3.7±0.6 cm and during error trials: 3.3±0.4 cm, *N*=4 rats, *P*=0.6). Thus, differences between self-initiated and uncued task performance are unlikely to result just from variability or changes of animal position in the training box.

### Self-initiation modulates cortical tone-evoked responses

How might behavioural engagement modulate neural activity for task performance? Recent reports show that auditory cortex is important for various forms of acoustic behaviour in rodents^5^,^39^,^40^,^41^. Moreover, we previously showed that either cholinergic or the noradrenergic modulation produced plasticity within the rat auditory cortex that could improve behavioural performance on this task for hours to weeks^4^,^29^. As the pure tone stimuli used in our task are highly processed by subcortical stations before reaching the cortex, the auditory cortex might encode the context dependence or behavioural significance of these sounds. Supporting this hypothesis, we found that neural activity in the auditory cortex was required for this behaviour. Bilateral muscimol infusion into the auditory cortex substantially impaired performance, whereas the same animals were unimpaired following saline infusion (Supplementary Fig. 3).

To determine how self-initiation modulates neural activity for improving sensory perception, we performed single-unit recordings from the auditory cortex of behaving rats chronically implanted with tetrode microdrives^5^,^32^,^42^. We recorded spiking activity from 227 neurons in 6 rats, including 117 units that were monitored during consecutive blocks of self-initiated and uncued trials. The advantage of this comparison is that the external context, the motor output, and the significance and value of tones are the same in both cases, allowing us to isolate neural processes that may be recruited during behavioural engagement in the self-initiated trials.

**Figure 2:**
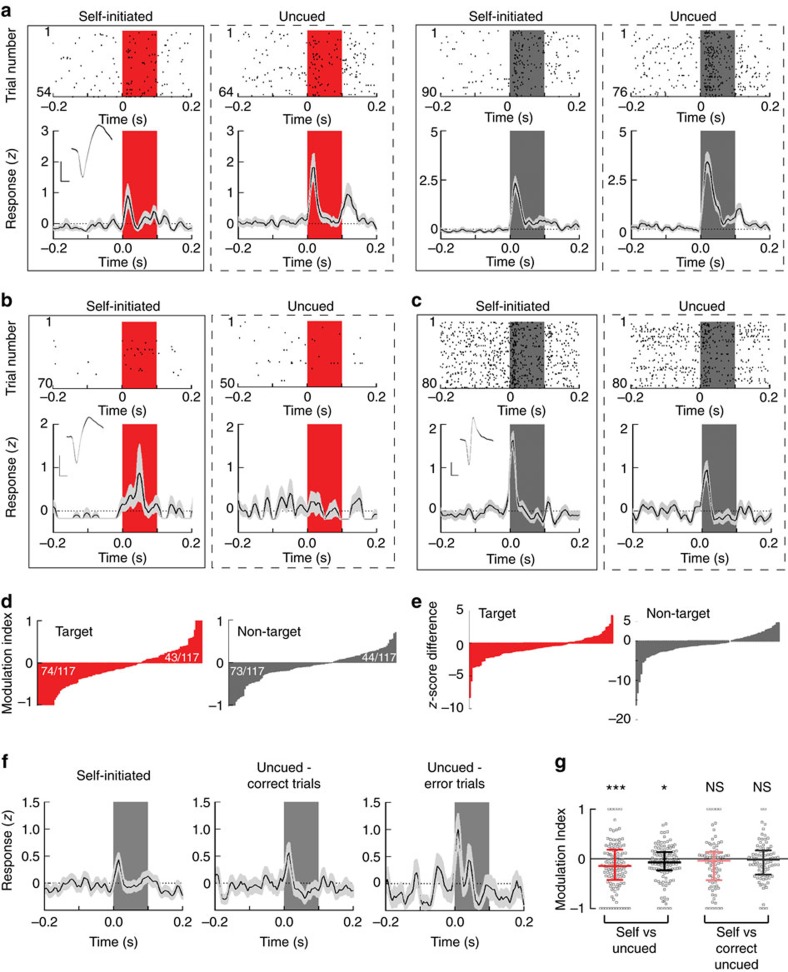
Self-initiation bidirectionally modulates evoked responses in the auditory cortex. (**a**) Example recordings from an isolated neuron in the auditory cortex. Left inset, spike waveform average and s.e.m. (grey shadow); horizontal scale bar, 0.4 ms and vertical scale bar, ∼40 μV. Raster plots (top) and *z*-score PSTHs (bottom) of recordings performed during uncued and self-initiated trials, aligned either to target tone onset (red bar) or to non-target tone (gray bar). For this example cell, responses to target and non-target tones were suppressed during self-initiated trials. (**b**) Example recordings from a cortical neuron for which responses to target tones were enhanced during self-initiated trials. (**c**) Example recordings from an isolated neuron in the auditory cortex for which responses to non-target tones were enhanced during self-initiated trials. (**d**) The distribution of the modulation index calculated for all 117 recorded neurons during target or non-target presentation, ordered in an ascending manner from left to right. Compared with responses during uncued trials, target-evoked responses were suppressed in 74/117 (63.2%) neurons and enhanced in 43/117 neurons. Non-target evoked responses were suppressed in 73/117 (62.4%) and enhanced in 44/117 neurons. (**e**) The z-score difference shows similar trends, where 68.7% of neurons had smaller target response *z*-score during self-initiated trials and 65% of neurons had smaller non-target response *z*-score during self-initiated trials. (**f**) Example cell *z*-score PSTHs during self-initiated trials and during correct uncued trials as well as error uncued trials. Grey bar, non-target tone. (**g**) The distribution of modulation indices for target and non-target frequencies, calculated for ‘Self' versus ‘Uncued' trial and for ‘Self' versus ‘Correct Uncued' trials. Error bars are s.e.m.

We first examined whether self-initiation affected tone-evoked responses (Fig. 2). For comparison across units, we normalized responses by calculating z-scores for all trials aligned to either target or non-target tone onset, and considered the ‘evoked response' as the peak *z*-scored firing rate up to 100 ms after tone onset (Fig. 2a–c). We found that, for the same units, evoked responses were different in the self-initiated versus uncued versions of the task. In some cells, the evoked response to tones was lower during self-initiated trials than during uncued trials (Fig. 2a, left; *z*-scored response to target was 0.8±0.3 during self-initiated trials and 1.8±0.4 during uncued trials, *P*=0.001, Student's unpaired two-tailed *t*-test; Fig. 2a, right, *z*-scored responses of the same unit to non-targets decreased from 3.4±0.5 during uncued trials to 2.1±0.3 during self-initiated trials, *P*=0.0003). In some other cells, however, evoked responses were higher during self-initiation (Fig. 2b, *z*-scored target responses increased from −0.2±0.0 during uncued trials to 0.9±0.6 during self-initiated trials, *P*=0.02; Fig. 2c, *z*-scored responses of a different unit to non-targets increased from 0.9±0.2 during uncued trials to 1.6±0.2 during self-initiated trials, *P*=0.0001).

To determine how self-initiation modulates evoked responses at the population level, we calculated a modulation index between ‘Self' and ‘Uncued' evoked responses: (*R*_self_−*R*_uncued_)/(*R*_self_+*R*_uncued_), where *R* is the firing rate. For the majority of cells, (74/117 units or 63.2%), self-initiation decreased the evoked response to targets (leading to negative modulation indices), a proportion similar to the suppression detected during the transition from passive to active listening in a different auditory behaviour^32^. For the remaining 43/117 cells, the evoked response was larger during self-initiated trials, represented by positive modulation indices (Fig. 2d, left). Confirming that the majority of neurons had suppressed responses to target tones during self-initiated trials, the median modulation index was negative and significantly different from zero (Fig. 2g, median modulation index: −0.14, interquartile range: −0.42 to 0.18, *n*=117 cells, *P*=0.003, Student's one-sample two-tailed *t*-test). Self-initiation similarly affected responses to non-target tones: evoked responses were suppressed in 73/117 neurons and enhanced in 44/117 neurons (Fig. 2d, right). For the majority of cells, most non-target evoked responses were also suppressed during self-initiation, leading to negative modulation index values (Fig. 2g, median modulation index: −0.07 interquartile range: −0.23 to 0.14, *n*=117 cells, *P*=0.003). We observed similar modulation patterns when we plotted the difference in z-score values between responses during ‘Self' and ‘Uncued' trials, indicating that the modulation of evoked responses by trial self-initiation does not result from global changes in spontaneous neuronal activity between the two versions of the task (Fig. 2e). Movement during behavioural trials did not significantly contribute to the modulation of evoked responses at the population level (Supplementary Fig. 4). For the majority of neurons, the correlation coefficient between target or non-target evoked response and the *x*- or *y*-motion was small and not significant (only 8/32 neurons had significant correlation between the evoked responses and *x*, *y*-movement preceding self-initiation).

We wondered whether the improved performance during self-initiated trials related to the modulation of evoked cortical responses. To examine this, we compared the evoked responses during self-initiated trials with the evoked responses during correct uncued trials. Interestingly, evoked responses during correct uncued trials resembled evoked responses during self-initiated trials (Fig. 2f). At the neuronal population level, the median modulation index between self-initiated trials and correct uncued trials was not significantly different from zero for target evoked responses (Fig. 2g, median modulation index: −0.03, interquartile range: −0.4 to 0.1, *n*=117 cells, *P*=0.07, Student's one sample, two-tailed *t*-test) or non-target evoked responses (Fig. 2g, median modulation index: −0.01, interquartile range: −0.3 to 0.2, *P*=0.2). Thus, the magnitudes of evoked responses are adjusted in a manner that predicts successful auditory detection and recognition. Therefore, it appears that self-initiation improved performance by recruiting brain states that are optimal for behavioural engagement or for stimulus expectation.

### Self-initiation controls cortical auditory receptive fields

We next asked whether, in the same neurons, targets and non-target responses were modulated in the same direction—that is, responses to targets and non-targets were both enhanced or both suppressed in individual units (Supplementary Fig. 5). Co-suppression of both target and non-target responses was observed in 50/177 recordings (42.7%; Supplementary Fig. 5a, lower left quadrant). In 21 other recorded neurons, self-initiation increased responses to both targets and non-targets (Supplementary Fig. 5a, upper right quadrant). For the other 47 cells, self-initiation differentially affected target and non-target tones, such that one set of responses was enhanced while responses to the other stimulus category was reduced (Supplementary Fig. 5a, upper left and lower right quadrants).

**Figure 3:**
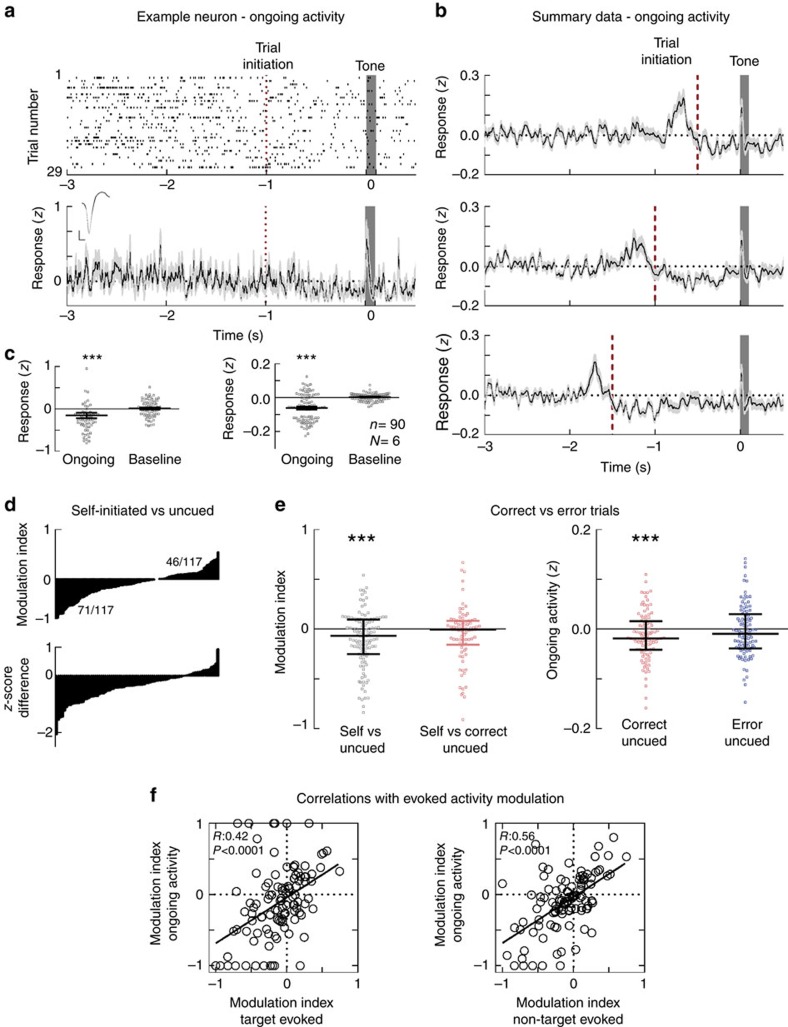
Self-initiation decreases ongoing activity. (**a**) Example single-unit recording from an auditory cortical neuron during self-initiated trials. Top: spiking activity for 29 trials. Spike trains were aligned to tone onset (grey bar represents all tones, target and non-targets). Bottom: *z*-score PSTH of the same recording. Inset shows the waveform for this example neuron. (**b**) Mean *z*-scores during self-initiated trials for all recorded cells. Trials were aligned to tone onset and self-initiation occurred at 0.5 (top), 1 (middle) and 1.5 (bottom) seconds before tone (dashed red line). *Z*-score values were smoothened with a factor of 7. (**c**) Left: the distribution of ongoing activity *z*-scores for the example neuron in (**a**). Right: summary data for the ongoing activity *z*-scores. (**d**) Top: the distribution of indices that measure modulation of ongoing activity by trial initiation (difference over sum of the ongoing firing rate during ‘Self' and during ‘Uncued', 500 ms before tone onset), for all 117 recorded neurons in the auditory cortex. Compared with uncued trials, ongoing activity was suppressed in 71/117 (60.6%) of neurons following self-initiation. Bottom: cell-by-cell *z*-score differences between ‘Self' and ‘Uncued' trials, calculated for the 500 ms of ongoing activity preceding tone onset. The *z*-score difference ranged between −2.06 and −0.003, and had a mean value of −0.5. (**e**) Left: the modulation indices calculated between ‘Self' and ‘Uncued' trials, and between ‘Self' and ‘Correct Uncued' trials (red,). Right: ongoing activity *z*-score values for correct uncued trials and for error uncued trials. (**f**) Left: correlation between the modulation index for responses to target and the modulation index for ongoing activity. Right: correlation between the modulation index for responses to non-target tones and the modulation index for ongoing activity. Error bars are s.e.m.

Surprisingly, these changes in target versus non-target tones during self-initiated trials could transform frequency tuning profiles of these neurons, including the best frequency. We measured frequency tuning of each cell during self-initiated (solid lines) and uncued (dashed lines) trials, fitting Gaussians to parametrize the peak and width of auditory cortical frequency tuning profiles (Supplementary Figs 5b and 6). When both target and non-target responses were similarly affected, tuning curve amplitudes were either increased or decreased during self-initiation. In contrast, when target and non-target responses were differentially modulated, this could sharpen or broaden tuning curves (that is, increasing or decreasing the width of the Gaussian fits). These changes in cortical frequency tuning can be observed in the example cells shown in Supplementary Figs 5b and 6: self-initiation sharpens the tuning profile either by increasing the response at a specific sound frequency (left) or by suppressing responses for most but not all sound frequencies (middle and right). To quantify this change in the sharpness of neuronal receptive fields, for each cell we aligned the tuning profiles to the best frequency and normalized them to the best frequency response (Supplementary Fig. 7a). The area under the curve calculated for the best frequency aligned plots is inversely correlated with how sharp the tuning profile of each cell is. For the example cells in Fig. 3b (from left to right), the area under the curve is 1.9, 1.2 and 1.4 during self-initiated trials and 2.3, 2.1 and 1.8 during uncued trials.

On average across the population of recorded neurons, self-initiation decreased the width of tuning curves (Supplementary Fig. 5c, left: mean self-initiated *σ* 5.6±1.4 octaves, uncued *σ* 12.5±3.0 octaves, *n*=41 cells, *P*<0.02, Student's paired two-tailed *t*-test), increased the dynamic range measured as the distance between the tuning curve maxima and minima (Supplementary Fig. 5c, middle: 0.7±0.0 during ‘Self', 0.6±0.0 during uncued trials, *n*=41, *P*<0.005), increased the sharpness of best frequency-aligned tuning profiles (Supplementary Fig. S7b, area under the curve for self-initiated trials was 0.7±0.07 and for uncued trials was 0.9±0.05, *n*=41, *P*<0.04), and improved the neuronal *d*′ values between targets and non-targets (Supplementary Fig. 5c, right: mean *d*′ for self-initiated trials was 4.1±1.1 and for uncued trials was 2.8±1.9, *n*=41, *P*<0.04).

To determine whether behavioural performance depends on the shape of cortical tuning profiles, we separately looked at how tuning during correct uncued trials compares with tuning during self-initiated trials. For some cells, tuning during correct uncued trials had an intermediate shape between self-initiated and uncued profiles (Supplementary Fig. 7b, left). At the population level, we find that tuning curves are equally sharp during correct uncued and self-initiated trials (Supplementary Fig. 7, the area under the curve for the best frequency-aligned tuning curves of correct uncued trials was 0.7±0.07, not significantly different from self-initiated trials).

To ask whether changes in evoked response magnitude and in receptive field structure are specific to the mode of trial initiation, as well as to exclude the possibility that the observed changes in receptive field structure resulted from degradation of the recording over time, we examined neural activity during a sequence of three consecutive sessions: uncued—self-initiated—uncued. For the cell in Supplementary Fig. 5d, the magnitude of evoked responses was comparable between the two uncued sessions but different during the self-initiated session (Supplementary Fig. 5d, *z*-score of evoked responses was similar between the two sessions of uncued trials: 0.5±0.2 for ‘Uncued 1'and 0.9±0.2 for ‘Uncued 2', and different for ‘Self' trials: −0.1±0.1, *n*=46 trials, *P*=0.005, one-way ANOVA and Dunnett's multiple comparison test). Similarly, the tuning profiles for this cell were comparable between the two uncued sessions but were sharper during the self-initiated session (Supplementary Fig. 5e, ‘Uncued 1' *σ*: 6.1 octaves, ‘Self' *σ*: 3.4 octaves, ‘Uncued 2' *σ*: 7.8 octaves). At the population level, evoked responses were highly similar between the two ‘Uncued' sessions, but decreased during the ‘Self' session (Supplementary Fig. 5f, ‘Uncued 1' *z*-score: 0.4±0.1, ‘Self' *z*-score: 0.1±0.0, ‘Uncued 2' *z*-score: 0.4±0.1, *n*=25 recordings, one-way ANOVA and Dunnett's multiple comparison test). Taken together, these data show that self-initiation induces a rapid and flexible restructuring of receptive fields related to improved behavioural performance.

### Self-initiation regulates cortical ongoing activity

It has been previously shown that cortical responses to visual and somatosensory stimuli correlate with patterns of spontaneous activity such as the ‘up' and ‘down' cortical states^13^,^43^,^44^,^45^. In addition to modulations of spiking during tone presentation, we also observed that self-initiation decreased the ongoing activity prior to tone onset (Fig. 3a, ongoing activity for an example neuron; top, tone-aligned raster plot for this cell; bottom left, tone-aligned *z*-score peristimulus time histogram (PSTH) and inset with waveform). At the population level, ongoing activity was gradually suppressed after self-initiation (Fig. 3b, *z*-score values progressively decreased from the time of self-initiation and were lowest before tone onset). The decrease in ongoing activity during the 0.5 s interval before tone (between −0.5 and 0 s before tone), was significant compared with a 0.5 s baseline measured before trial initiation (between −2.5 and −2.0 s), for both the example neuron (Fig. 3c, left: trial-to-trial *z*-scores for the ongoing activity decreased from 0.01±0.02 before self-initiation to −0.15±0.06 before tone, *n*=63, *P*=0.0007, Wilcoxon matched-pairs signed-rank test) and at the population level (Fig. 3c, right: mean *z*-score for ongoing activity:−0.061±0.008, mean *z*-score for baseline activity: 0.003±0.002, *n*=117 cells, *P*<0.0001). We calculated the modulation of spiking due to self-initiation versus uncued trials for the 0.5 s of ongoing activity preceding the tone onset. Self-initiation suppressed spontaneous activity in 71/117 (60.6%) neurons (Fig. 3d top and Fig. 3e, median modulation index: −0.07, interquartile range: −0.25 to 0.09, *n*=117 cells, *P*<0.0001, one-sample two-tailed *t*-test). Similarly, the difference in trial-by-trial ongoing activity *z*-scores between self-initiated and uncued trials was negative in the majority of neurons, confirming that these changes in spontaneous activity result from trial specific modulations rather than from global changes in neuronal firing (Fig. 3d, bottom). We calculated modulation indices between self-initiated and correct uncued trials for each cell and found that they were similar (Fig. 3e, left: median modulation index between ‘Self' and correct uncued trials was −0.005, with an interquartile range between −0.157 and 0.081, *n*=117 cells, *P*=0.07, Student's one-sample two-tailed *t*-test). Moreover, ongoing activity was significantly suppressed during correct but not error uncued trials (Fig. 3e, right: for correct uncued trials the median *z*-score was −0.019 with interquartile range between −0.041 and 0.015, *n*=117, *P*=0.005, Student's one-sample two-tailed *t*-test; for error uncued trials the median *z*-score was −0.009, with an interquartile range between −0.038 and 0.029, *P*=0.5). The magnitude of ongoing activity suppression varied from cell to cell and was significantly correlated with the modulation of target and non-target evoked responses in the same units (Fig. 3f). The suppression of ongoing activity did not depend on the movement of the animal during the self-initiated trials (Supplementary Fig. 8). This suggests that self-initiation regulates the rate of ongoing activity, to more precisely control sensory-evoked responses and boost signal-to-noise ratios.

### Optogenetic disruption of auditory perception

**Figure 4:**
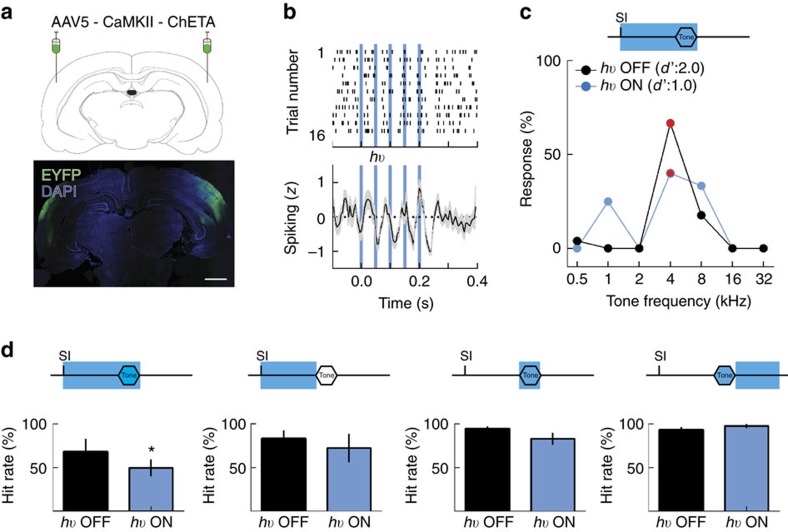
Cortical noise injection before and during tone presentation disrupts behavioural performance. (**a**) Top: expression of the ChETA-EYFP construct was induced by injecting a virus bilaterally in the auditory cortex of trained rats. Bottom: appropriate expression was confirmed by immunohistochemistry for the GFP protein (green). Scale bar, 2 mm. (**b**) Top: raster plot for an example neuron recorded during optical stimulation (blue lines) of the auditory cortex in a freely behaving rat. Bottom, *z*-score PSTH for the example recording above shows that light stimulation can drive activity in the auditory cortex. (**c**) Top: schematic of the stimulation protocol: a train of 5 ms blue light pulses was delivered bilaterally or unilaterally at 20–30 Hz in the auditory cortex during the behavioural trial. Self-initiation triggered the light train, which lasted until tone offset. The ‘light ON' trials (blue) were interleaved at a 1:5 ratio with ‘light OFF' trials (black). Bottom: compared with control values, hit rate decreased during full-trial activation of the auditory cortex (blue line). (**d**) Top: schematic of the stimulation protocols. Bottom: hit rate performance. Error bars are s.e.m.

To investigate the role of ongoing activity patterns in sensory perception, we used optogenetics to manipulate neuronal activity in the auditory cortex. In six adult rats, we injected adeno-associated virus (pAAV5-CaMKII-ChETA-EYFP) bilaterally in the auditory cortex for expression of the channelrhodopsin-2 ‘ChETA' variant under the CaMKII promoter^46^. Animals also had either optical fibres or optical microdrives implanted in the auditory cortex. Location of viral injections and fibre implantation was established during surgery by multiunit electrode mapping of the auditory cortex and confirmed with immunohistochemistry at the end of each experiment (Fig. 4a).

During behavioural testing in the injected rats, we delivered pulses of blue light at 20 Hz via the optical implants. We recorded from the auditory cortex during this procedure, to determine how optical stimulation affected cortical activity (Fig. 4b, example neuron recorded during stimulation with 5 ms blue light pulses). Optical stimulation began at self-initiation and occurred either throughout the trial (starting at self-initiation and ending at tone offset) or only during the ongoing activity period (ending just before tone onset), or during the tone, or only post-tone (for one second after tone-offset). Optogenetic stimulation of the auditory cortex was capable of disrupting normal patterns of activity, as can be observed during ongoing activity (Supplementary Fig. 9, left; *z*-score for control trials was significant lower than 0: −0.01±0.00, *n*=30 cells, *P*=0.04, Student's one-sample two-tailed *t*-test; *z*-score for optogenetically disrupted trials was not significantly smaller than 0: −0.006±0.00, *n*=30 cells, *P*=0.4) and during evoked activity (Supplementary Fig. 9, right; the mean modulation index between optically disrupted and control trials was 0.08±0.02, *n*=30 cells, *P*<0.008, one sample *t*-test). Stimulation during ongoing and evoked periods decreased hit rates (Fig. 4c, example rat hit rate was 67.3% during light OFF trials 40% and during light ON trials, *n*=26 trials, *P*=0.04, Fisher's exact test; Fig. 4d, summary data: hit rate was 68.1±15.0 during control, light OFF trials and 50.0±9.6 during light ON trials, *N*=6 rats, *P*=0.03, Student's ratio paired two-tailed *t*-test). In contrast, stimulation only during the ongoing activity period (light OFF hit rate was 83.0±9.8 and light ON hit rate was 72.6±16.3, *P*=0.3), the tone period (light OFF hit rate was 94.2±3.0 and light ON hit rate was 83.0±7.0, *P*=0.1) or post-tone period (light OFF hit rate was 92.8±3.8 and light ON hit rate was 97.6±2.3, *P*=0.4) did not significantly affect responses to targets (Fig. 4d). Thus, disrupting both ongoing and evoked activity during self-initiated trials impaired behavioural performance.

## Discussion

Here we show that self-initiated behavioural engagement bidirectionally modulated cortical responses to behaviourally meaningful sensory cues. The majority of responses were rapidly but flexibly suppressed, but a smaller percentage of neurons showed enhanced responses during self-initiation. These modulations were independent of movement or the position relative to the speaker. This suggests that there might be an increase in the signal-to-noise ratio at the population level that favours sound detection and recognition. In cells where responses to both target and non-target tones were suppressed, we observed a decrease in the amplitude but no significant change in the structure of tuning profiles. This adaptive stimulus-generalized suppression could limit the spread of activation within the auditory cortex and therefore could promote sparse coding and increase separation between the representations of stimuli in different cells. In other cells, responses to target and non-target tones were modulated in opposite directions, leading to significant sharpening of frequency tuning profiles. Surprisingly, we did not observe an overall increase in the representation of the target tone—instead, one of the tones (either target or one of the non-target tones) gained stronger representation compared to the rest of the tones. Together, the significant changes in receptive field structure lead to better separation between target and non-target responses. Importantly, these modulations could be caused by changes in head orientation, as we presented the auditory stimuli via a free-field speaker. However, these changes in receptive fields resemble modulations observed in the visual cortex of primates, where attention increases the response of a cell to the preferred stimulus and decreases its responses to non-preferred stimuli^47^,^48^,^49^. In the rat auditory cortex, stimulus expectation can also lead to sharpening of tuning profiles^5^. Thus, self-initiated engagement in behavioural tasks might recruit brain states that are optimal for stimulus processing by activating neuronal mechanisms that are involved in attention and in the anticipation of sensory inputs.

One of the main differences between self-initiated and uncued trials was that in the former task rats learned to expect a tone 0.5, 1 or 1.5 s after self-initiation. During this interval, ongoing activity in the auditory cortex was significantly suppressed in the majority of recorded neurons. This suppression correlates with behavioural performance and is independent of movement. Our data agree with recent finding that show that certain arousal states promote membrane hyperpolarization in the auditory cortex and optimize perceptual performance^36^. Although in our studies, movement did not play a significant role in the modulation of ongoing or evoked activity during self-initiated trials, this is likely due to the lack of significant movement in the behaviour chamber, while animals waited for the tone or while the tone was presented. Thus our findings do not contradict recent studies showing that movement controls activity in the auditory cortex^30^,^36^.

In other sensory areas such as the visual cortex, naturally occurring fluctuations in arousal level or induced arousal can also suppress ongoing activity^47^. We found that changes in ongoing activity during engagement were significantly correlated with changes in non-target tone evoked activity. This is consistent with previous findings in the visual and somatosensory cortex that natural fluctuations in the membrane potential (for example, during up and down states) are positively correlated with the stimulus-evoked response^13^,^44^,^50^. Even in absence of two-state dynamics such as these, spontaneous and evoked activity levels in auditory cortex can be correlated^17^. Thus, changes in the rate of ongoing activity reflect network dynamics that control cellular excitability. However, there is evidence in both the visual and the somatosensory cortex that suppression of ongoing activity could result in increased signal-to-noise ratio^49^,^51^. It is possible that during self-initiated trials, suppression of spontaneous activity serves both purposes: to decrease neuronal excitability in most of the cells and to selectively increase the signal-to-noise ratio in other cells (those units that show increased tone-evoked responses following self-initiation). As some of the cells had differentially modulated responses to target and non-target tones, the behavioural control of spontaneous activity could selectively modulate responses to specific tones.

Suppression of ongoing activity by cortical states contributes to perception. In gerbils, suppression of ongoing activity before tone onset has been hypothesized to improve stimulus detection by reducing the detection threshold^14^. In our study we established a causal link between suppression of ongoing activity following self-initiation and behavioural performance using optogenetic stimulation of the auditory cortex during behaviour. In addition to increasing the mean firing rate, this manipulation may have also increased noise correlations in the auditory cortex and introduced artificial patterns of activity. Thus, the optogenetic stimulation disrupted normal modulation patterns in the auditory cortex and showed that modulation of both evoked and ongoing activity are necessary for improved performance during self-initiated trials.

What are the neuronal mechanisms underlying the changes in spontaneous and evoked activity observed during self-initiated trials? Several neuromodulatory systems are engaged during behavioural performance and contribute to the level of alertness, attention and motivation of the animal. The cholinergic and the noradrenergic innervation of the cortex is of particular interest for the behavioural task we described as they can lead to either suppression or enhancement of cortical activity^4^,^24^,^26^,^29^,^40^,^52^. In addition to these, frontal cortical areas where neuronal activity is modulated during the ‘waiting' time between self-initiation and stimulus onset^53^ could provide top–down control of spontaneous and evoked activity in the auditory cortex^30^.

We found that behavioural self-engagement can recruit cortical states for optimal sensory perception by modulating evoked and ongoing activity in the auditory cortex to increase signal-to-noise ratio at the level of individual neuron receptive fields as well as at the population level. When these patterns of cortical modulation are disrupted behavioural performance during self-engagement decreases, indicating that precise control of ongoing and evoked activity in the auditory cortex is required for sensory perception.

## Methods

### Behavioural training

All animal procedures were performed in accordance with National Institutes of Health standards and were approved by the New York University School of Medicine Institutional Animal Care and Use Committee.

The behavioural task used here was similar to that we used previously^4^,^29^,^54^ with the addition that animals could self-initiate trials during some testing sessions^54^. Animals were trained on a go/no-go task to nosepoke in response to a target tone frequency for a food reward in 9" × 10" × 12" operant conditioning chambers (Med Associates, Inc.). Each chamber contained a speaker (on the right wall) calibrated across frequencies at 70 dB SPL, a food dispenser on the left wall and three nose poke ports (two on either side of the food dispenser and one on the wall opposite). Each chamber was placed in a larger wood enclosure and insulated with foam. The measured background noise in each chamber was <30–40 dB SPL.

Twenty-five adult, female Sprague–Dawley rats were used in these behavioural studies. Animals were food restricted to maintain the weights at 80–85% of their initial pre-training weights. First, animals were shaped with two days of training to nosepoke for one food pellet. Next, rats were trained to nosepoke within 2.5 s after a target tone was played (for 23 rats the target tone was 4 kHz and for two rats the target tone was 16 kHz). When the rats had hit rates of >50%, six non-target tones were introduced (0.5–32 kHz at one octave intervals excepting the target frequency) and animals were trained to hit rates >65%. Target and non-target pure tones were 100 ms in duration presented in a pseudo-random order at 70 dB SPL. For correct trials, each trial ended at either the time of food pellet delivery (hit trials for targets) or 2.5 s after the tone (correct reject trials for non-targets). On error trials, failure to respond (miss trials for targets) as well as incorrect responses (false alarm trials for non-targets) were punished with a time-out of 7 s before the next trial began. Random nose pokes were punished with time-out as well.

In some blocks of behavioural testing, rats self-initiated the trials by nosepoking in a different port than the ‘response' port. After 0.5–1.5 s, either a target or non-target tone was played. In other blocks of trials (‘Uncued'), the trials started 6–10 s after the end of the previous trial or after the time-out interval. Overall, the trial-to-trial interval for uncued trials was 6–10 s when the previous response was correct or 13–17 s when the previous response was incorrect. The trial-to-trial interval increased above these values when the animal made random nose pokes.

For the detection task, the amplitude of target and non-target frequencies was varied randomly between 20 and 80 dB SPL (at 10 dB SPL intervals). For testing performance on the narrowband version of the task, the non-target frequencies were changed to spectrally closer (1/6 of an octave), whereas the target frequency remained the same. In the experiments where the sounds were presented via headphones, we built special adapters from copper mesh and grip cement that were then implanted over both ear canals of the rats. After recovery, commercial headphones with a 20 kHz cutoff frequency were connected to the sound generators of the MedAssociates chambers and then fitted in the adapters.

In five trained adult rats, we bilaterally implanted cannulas above the auditory cortex at the following stereotactic coordinates from Bregma: −5.5 anterior-posterior (AP), 6.5 medial-lateral (ML), −1.5 dorsal-ventral (DV). Following recovery from surgery, the rats were trained to recover behavioural performance and were tested as follows: before any injection, 20 min following injection of 2 μl muscimol solution (1 mg ml^−1^) in each cannula or 20 min following 2 μl saline injections in each cannula.

Behavioural performance was estimated with hit rate measurements (per cent of times the rats respond to the target frequency) and the discriminability index *d*′ (the difference in the *z*-scores for the distribution of responses to targets and for the distribution of responses to non-targets).

### Movement data collection and analysis

To monitor the movement of the rats in the behavioural chamber, we used video tracking to measure their position in the operant conditioning box during behavioural testing. An infrared camera (OptiTrack) was mounted on the ceiling of the wood enclosure. The camera was connected to the video tracking system (NeuroMotive), which recorded the position in *x* and *y* coordinates of a luminescent tag placed on the head of the animals, with a resolution of 640 × 480 pixels and a frame rate of 25 Hz. To calculate the movement during different behavioural trials, we calculated the difference in the x and y coordinates during Interval A (2 s before trial initiation, and from 3.5 to 1.5 s before tone during uncued trials) and during Interval B (the interval between self-initiation and tone, and 1.5 s before tone during uncued trials).

### Microdrive assembly and implantation

Eight tetrode Versadrives (Neuralynx) were assembled using 12.5 μm nichrome wire. Tetrodes were spun using the OpenEphys tetrode spinner (80 turns forward and 40 turns backwards) and were consolidated using a heat gun. Tetrodes were cleaned and electroplated in gold solution until their impedance was ∼0.2–0.5 MΩ (NanoZ electroplater, Neuralynx). For simultaneous recordings and optogenetic stimulations, optical microdrives were prepared, where a 200 μm optic fibre was inserted in the middle of the microdrive.

For implantation, six of the rats tested behaviorally were anaesthetized with ketamine/dexmedetomidine (75–100/0.5–1 mg kg^−1^, intramuscularly) A craniotomy was performed over the right auditory cortex (AP: −5.5 to −6.5 mm, ML: 6 to 6.5 mm). Primary auditory cortex (AI) was coarsely mapped by recording multi-unit responses with a tungsten electrode. After confirmation of location within AI, the microdrive was lowered down 0.9–1.1 mm and secured with bone screws and dental cement. Reference and ground electrodes were soldered to separate bone screws above the cerebellum. Rats were allowed to recover for ∼7 days after surgery.

### Neuronal data collection and analysis

Extracellular spiking data was collected with a 32 channel digital headstage and a neural signal processor (Blackrock Microsystems). Neuronal responses were recorded at 30 kHz, bandpass filtered at 0.25–5 kHz and artefacts rejected based on refractory period violations (inter-spike interval (ISI) <1 ms) and amplitude violations (>1–2 mV). The waveforms were sorted offline using a time-amplitude algorithm (nPlay Sorter). Spike trains were aligned using Neuroexplorer software either to the onset of all tones, to the onset of target tones alone or to the onset of non-target tones. For error trial analysis, we separated the ‘Uncued' trials in correct and error trials. The spike counts between −3 and 3 s were exported in 5 ms bins. Tetrodes were lowered by ∼75 μm at the end of each recording session.

Neuronal responses were analysed using Matlab (Mathworks). Changes in firing rate were quantified by calculating the trial-by-trial *z*-score for each 5 ms time bin of the tone-aligned spike trains, using the following formula: *z*=(*x*−*μ*)/*σ*, where *μ* represents the mean firing rate and *σ* represents the s.d. of the trial (3 s before tone onset). The modulation index for the evoked activity of each cell was calculated as the difference over sum of evoked firing rates *R* during self-initiated versus uncued trials: (*R*_self_−*R*_uncued_)/(*R*_self_+*R*_uncued_). We used a 15 ms window centred around the peak of the evoked response for calculating this modulation index. Similarly, the modulation index for the ongoing activity was calculated as the difference over sum of the firing rates during the 500 ms preceding the tone in self-initiated versus uncued trials.

Neuronal receptive fields were constructed by separating the evoked responses to each individual frequency tone (in the subset of recordings when we registered the timestamps of individual tone frequencies). We included in this analysis only those neurons that had a normal distribution of responses to different tone frequencies, as measured with the Shapiro–Wilk normality test (*α*=0.05). Based on these criteria, we excluded 24 cells from receptive field analysis. Other 52 neurons could not be included in this analysis, because the timestamps of different tone frequencies were not recorded separately. Gaussian curves were then fitted on the tuning profiles and the s.d. (in octaves) of each distribution was calculated. The dynamic ranges of the tuning curves were calculated as the differences between the maximum and the minimum normalized spiking responses. The neuronal *d*′ was calculated only for those cells that had a significant modulation of *z*-score values between the self-initiated and uncued trials (Student's *t*-test, *P*<0.05), as the difference between the *z*-score for non-targets and the *z*-score for target tones.

### Optogenetic stimulation

We injected bilaterally in the auditory cortex an AAV5 virus encoding ChETA (an optimized version of channelrhodopsin-2) under the CaMKII promoter (to limit expression to excitatory neurons). ChETA was fused with enhanced yellow fluorescent protein (EYFP), to allow for immunohistochemical confirmation. The virus was purchased from Addgene via the Penn Viral Core. The auditory cortex was targeted based on stereotactic coordinates (see above) and the location was confirmed electrophysiologically by multiunit recordings in anesthetized rats. For each hemisphere, we injected 1 μl of virus at two different sites in the auditory cortex. Following the injection, on each side of the brain a 200 μm fibre optic (Thor Labs) was implanted down to 1 mm below the brain surface and secured in place using Metabond and grip cement. We allowed two weeks for viral expression. During behavioural testing we connected a blue laser (400 nm) to the bilaterally implanted ferules. The light intensity at the tip of the fibre was ∼2 mW. The laser was triggered by a transistor-transistor logic (TTL) pulse sent by the Med Associate boxes when the animal self-initiated. The light ON trials were randomly interleaved with light OFF trials in a 1/5 ratio. When the laser was triggered, it delivered a 20 Hz pulse train for 0.5 s, until tone onset (in this experiment we only used 0.5 s delays between self-initiation and tone).

At the end of behavioural studies, rats were perfused with 4% paraformaldehyde, brains collected, cryoprotected in 30% sucrose and then sliced in 15 μm thin sections using a cryostat. We then performed immunohistochemistry using a rabbit anti-GFP antibody (1:500, Abcam) and Cy2-conjugated anti-rabbit secondary antibody (Jackson Immunoresearch). The sections were visualized using a Zeiss LSM 700 confocal microscope.

### Data availability

The data that support the findings of this study are available from the corresponding author upon reasonable request.

## Additional information

**How to cite this article:** Carcea, I. *et al*. Dynamics of auditory cortical activity during behavioural engagement and auditory perception. *Nat. Commun.* **8,** 14412 doi: 10.1038/ncomms14412 (2017).

**Publisher's note:** Springer Nature remains neutral with regard to jurisdictional claims in published maps and institutional affiliations.

## Supplementary Material

Supplementary Information Supplementary Figures

Supplementary Movie 1 Example rat performing the self-initiated version of the auditory go/no-go task

Supplementary Movie 2 Example rat performing the uncued version of the auditory go/no-go task

Supplementary Movie 3 Example performance with controlled delivery of acoustic stimuli via headphones
