# Supplementary material for: Dynamics of auditory cortical activity during behavioural engagement and auditory perception
Source: Nat Commun. 2017 Feb 8;8:14412. doi: 10.1038/ncomms14412 (PMC5309852; doi:10.1038/ncomms14412)
Supplement: Supplementary Information — Supplementary Figures [file ncomms14412-s1.pdf]

Supplementary Figure Legends

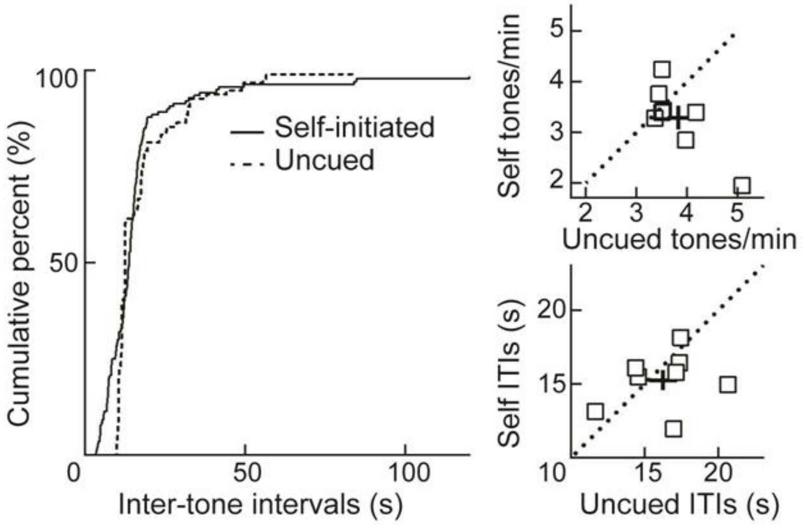

Supplementary Figure 1

Supplementary Figure 1: Self-initiation and uncued sessions have a similar number of trials

Left, example cumulative distribution of inter-trial intervals (ITIs) for consecutive self-initiated and uncued sessions in one example rat (average ITI: 18.1±2.3 seconds for self-initiated trials and 17.7±1.2 seconds for uncued trials, n= 201 trials, p=0.16, Mann-Whitney test).

Top left, summary data of ITI values during self-initiated (15.2±0.7 seconds) and uncued (16.2±1.0 seconds) behavioral sessions (N=8, p=0.3, Student's paired two-tailed t-test).

Top right, summary data showing the rate of tone presentation during self-initiated (3.3±0.2 tones/min) and uncued (3.8±0.2 tones/min) behavioral sessions (N=8 rats, p=0.2, Wilcoxon matched-pairs signed rank test).

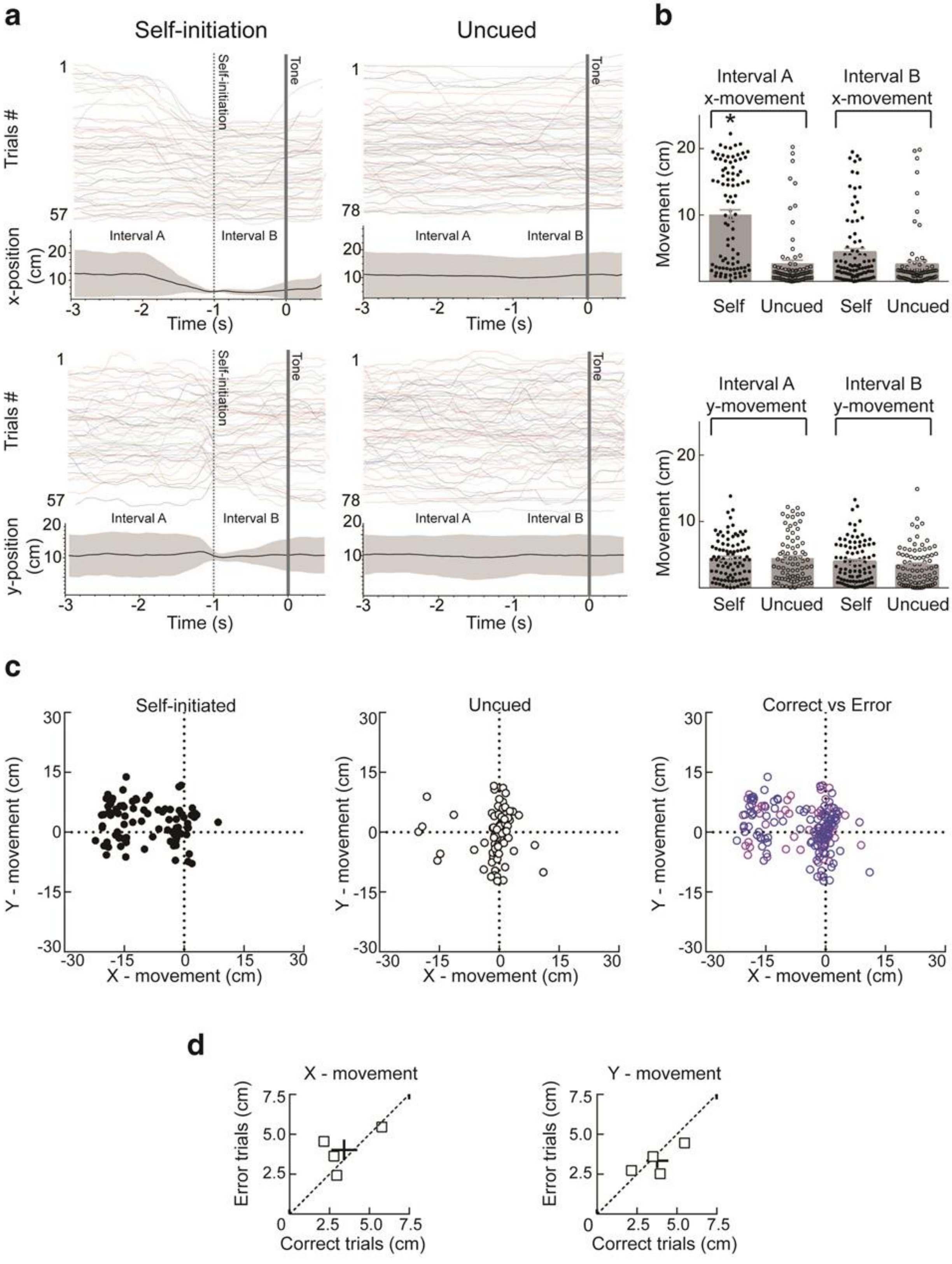

Supplementary Figure 2

Supplementary Figure 2: Movement does not impact behavioral performance

(**a**) Changes in the position within the behavioral box for one example rat during self-initiated (left, 1 second interval) and uncued (right) trials. The x (top) and y (bottom) coordinates obtained from video-tracking data were aligned to tone onset and revealed two intervals with different movement patterns during self-initiated trials. We defined 'Interval A' as the 2 seconds that preceded self-initiation and 'Interval B' as the time between trial self-initiation and tone. For uncued trials, where the trial start was not signaled by internal or external cues, 'Interval' A was the time between 3 to 1 seconds before tone and 'Interval B' was the time between 1 second and tone onset. Thus, the intervals were balanced between self-initiated and uncued trials. The x,y motion during uncued trials was minimal during both intervals. For self-initiated trials, the change in x,y position of the rat during 'Interval A' was substantial in part of the trials (colored lines), but was minimal between self-initiation and tone onset.

(**b**) Quantification of the motion during self-initiated (filled circled) and uncued (empty circles) trials. Motion was calculated as the difference in absolute values between position at the beginning and at the end of either Interval A or B. Top, motion in the x coordinate: motion during Interval A of self-initiated trials was 10.0±0.7 cm, significantly larger than movement during Interval B of self-initiated trials (2.7±1.5 cm), and also larger than movement during uncued trials (4.4±0.5 cm for Interval A and 2.5±0.5 cm for Interval B, n=91 self-initiated trials and n=78 uncued trials, $p<0.0001$, one-way ANOVA with Tukey's multiple comparison test). Bottom, motion in the y coordinate was similar for all intervals: 4.4±0.3 cm during Interval A of self-initiated trials, 4.4±0.3 cm during Interval B of self-initiated trials, 4.0±0.3 cm during Interval A of uncued trials and 3.4±0.3 cm during Interval B of uncued trials ($p=0.1$).

(c) Trial-by-trial movement in both x and y coordinates for the example rat in (a) during 'Interval A' of self-initiated (left), uncued (middle) and correct vs error trials (right). Correct (red) and error (blue) trials were similar in terms of motion in the x coordinate (6.8±0.7 cm for correct and 6.2±0.8 cm for error trials, n=96 correct and 73 error trials, p=0.5, Mann Whitney two-tailed test) as well as in the y coordinate (4.4±0.3 cm for correct and 4.3±0.3 cm for error trials, p=0.8).

(d) Summary data showing motion during 'Interval A' of correct and error trials for four rats. In the x coordinate (left), the motion during correct trials was 3.4±0.8 cm for correct trials and 4.0±0.6 cm for error trials (N=4, p=0.6, Wilcoxon matched-pairs two-tailed signed rank test). In the y coordinate (right), the motion during correct trials was 3.7±0.7 cm for correct trials and 3.3±0.8 cm for error trials (p=0.6). Error bars represent mean±SEM.

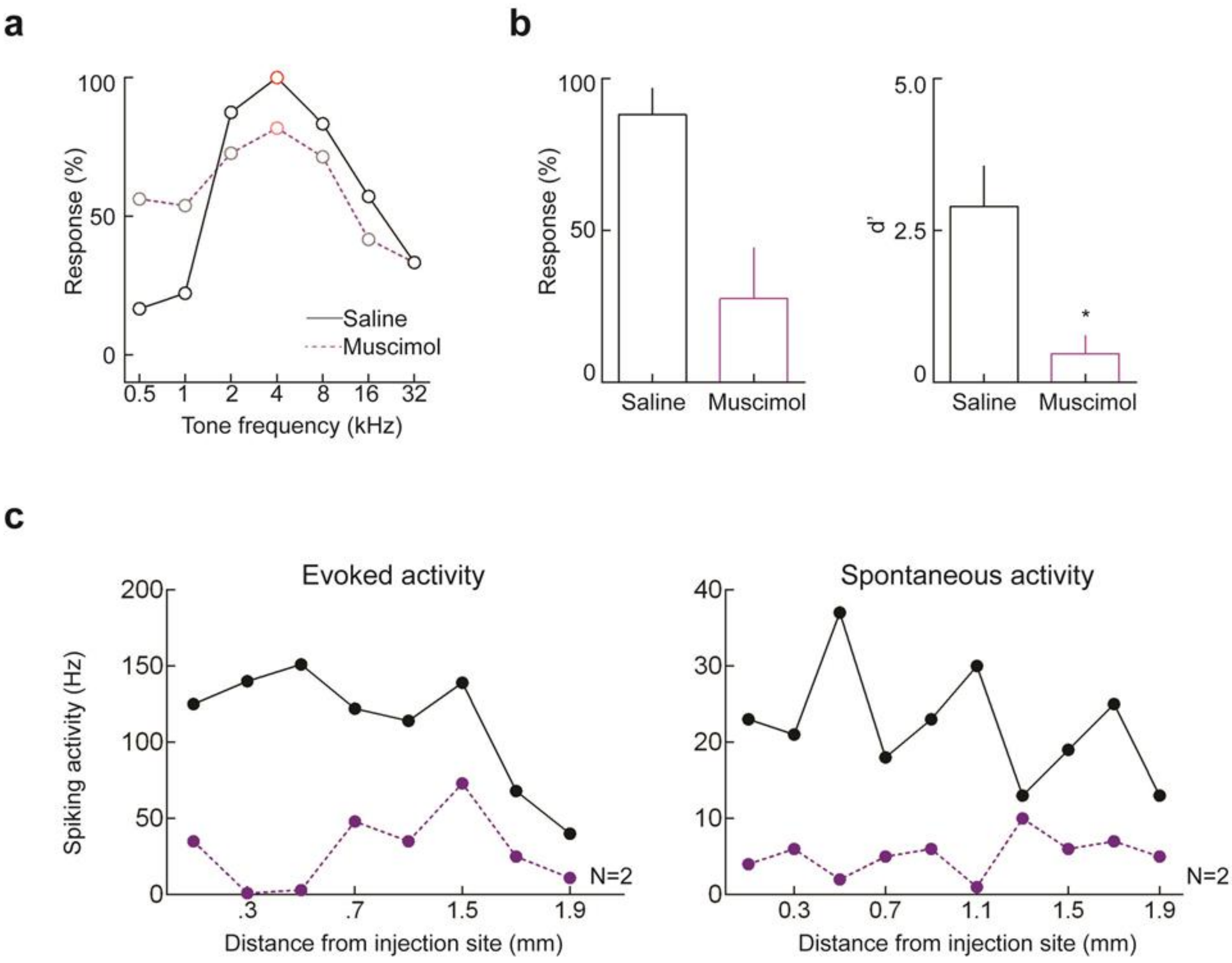

Supplementary Figure 3

Supplementary Figure 3: Auditory cortex is required for frequency recognition performance

(**a**) Example performance of a rat 20-80 minutes after 2 μl of either saline (black solid line) or 2 mg/ml muscimol (purple dashed line) was infused bilaterally in the auditory cortex.

(b) Summary data for hit rates (left) and d' values (left) for 5 rats injected with either muscimol or saline in the auditory cortex (hit rates: 88.1±8.7% for saline vs. 27.6±16.7% for muscimol,

N=5, p=0.04, Student's paired two-tailed t-test; d': 2.9±0.7 for saline and 0.5±0.3 for muscimol, p=0.04).

(c) Effects of muscimol on evoked (left) and spontaneous activity (right) up to 2 mm from the injection site (N=2).

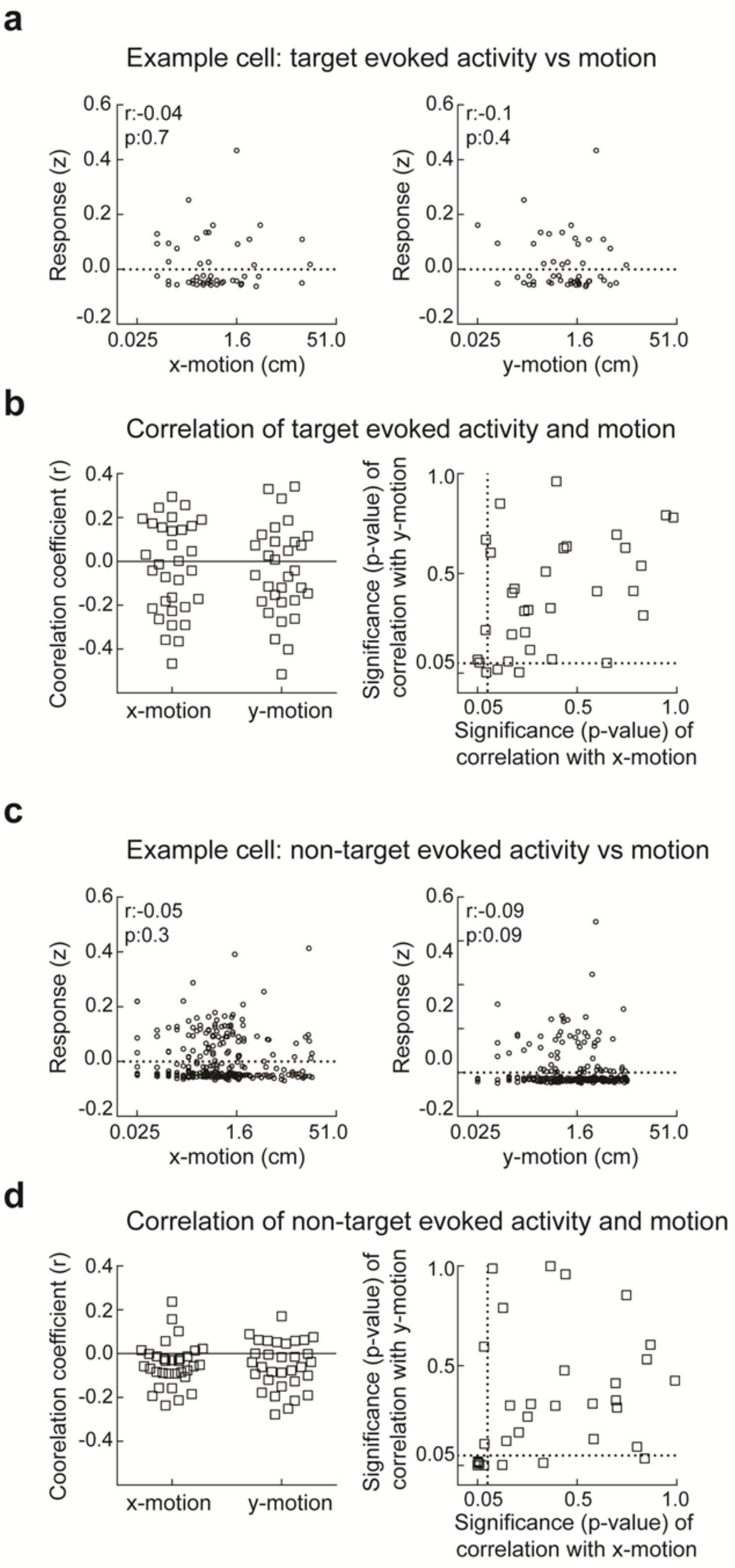

Supplementary Figure 4

Supplementary Figure 4: Evoked activity in the auditory cortex does not depend on pre-trial movement

(a) Example cell z-score values for target evoked activity as a function of trial-by trial movement during 'Interval A' in the x-coordinate (left) and y-coordinate (right).

(b) Spearman correlation coefficients (left) and p-values (right) for all 32 cells recorded. Only 8/32 cells showed significant correlation with either x- or y-motion or both.

(c) Example cell z-score values for non-target evoked activity as a function of trial-by trial movement during 'Interval A' in the x-coordinate (left) and y-coordinate (right).

(d) Spearman correlation coefficients (left) and p-values (right) for all 32 cells recorded.

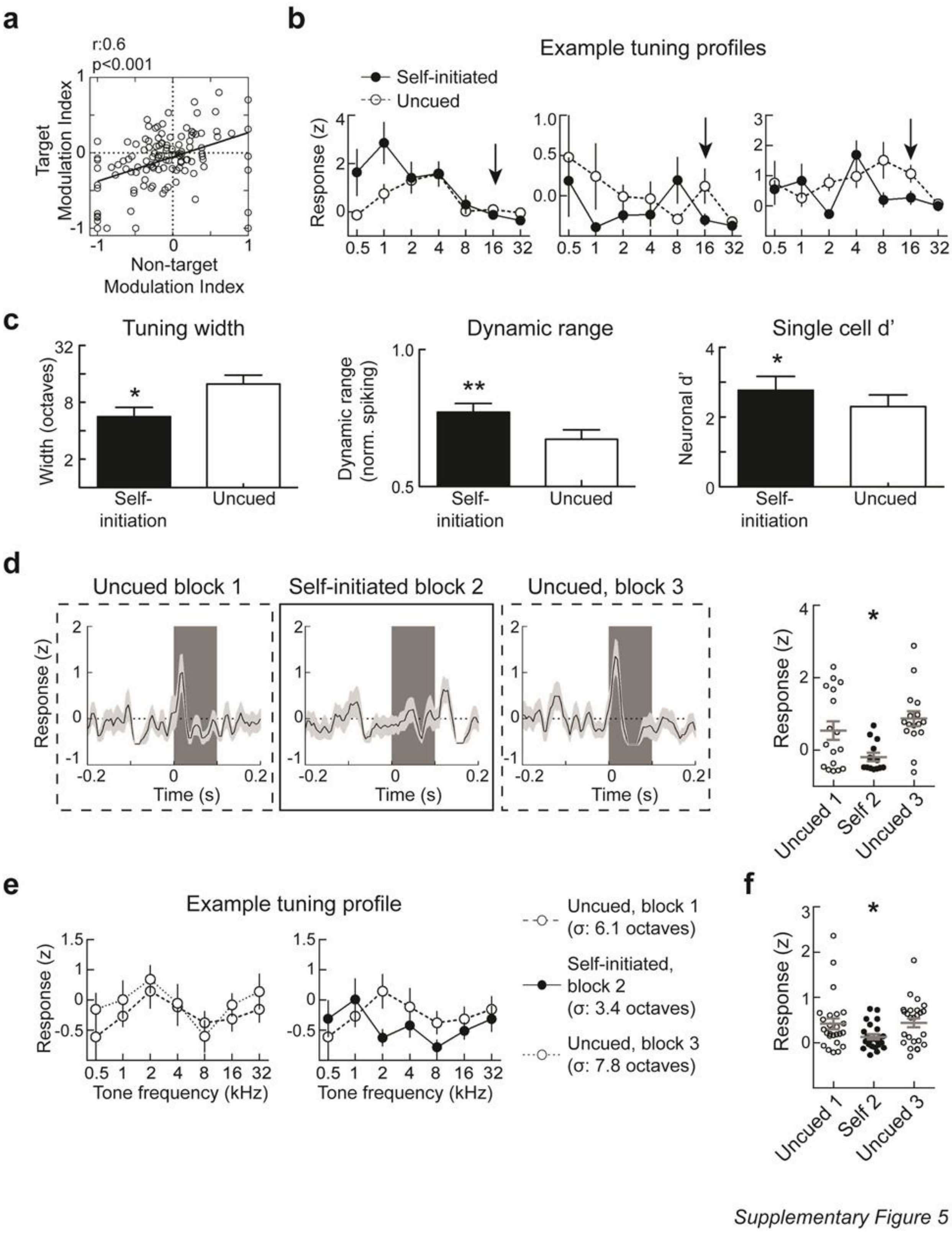

Supplementary Figure 5. **Self-initiation changes tuning curves and improves coding**

(**a**) Distribution of modulation indices for responses to target and non-target tones (r=0.4, p<0.0001, Spearman correlation).

(**b**) Example tuning profiles for neurons recorded during uncued (empty circles, dashed line) and self-initiated (filled circles, solid lines) conditions. Arrow, target frequency. Self-initiation increases the response to one frequency and decreases the response to the rest of the frequencies, thus sharpening the tuning curves.

(**c**) Left, population analysis showing decreased width (σ) of the Gaussian fit to tuning profiles during self-initiated trials compared to uncued trials. Mean 'self-initiated' σ is 5.6±1.4 and mean σ for uncued trials is 12.5±3.0, n=41 cells, p<0.02, Student's paired two-tailed t-test. Middle, at the population level, the dynamic range of the tuning profiles was higher during self-initiated trials (0.7±0.0) compared to uncued trials (0.6±0.0), p<0.005. Right, self-initiation significantly increased neuronal d'. Mean d' for self-initiated trials was 4.1±1.1 and for uncued trials was 2.8±1.9, p<0.04.

(**d**) Stable state-dependent differences between self-initiated and uncued responses. z-score PSTHs of an example neuron recorded during three consecutive behavioral blocks in the following sequence: uncued, self-initiated, uncued. The trials were aligned to tone onset (gray bar is tone). The cell had clear tone evoked responses during the first uncued session that were suppressed during the session of self-initiated trials but then recovered during the subsequent uncued session. Right: example cell mean z-score was 0.5±0.2 for 'Uncued 1', -0.2±0.1 for 'Self' and 0.9±0.2 for 'Uncued 2', n=46 trials, p=0.005, one way ANOVA, Dunnett's multiple comparisons test.

(e) Tuning profiles of the recordings in (**d**) show similar tuning between the two 'uncued' sessions (left) but sharper tuning during the session of self-initiated trials (right). 'Uncued 1' sigma: 6.1 octaves, 'Self' sigma: 3.4 octaves, 'Uncued 2' sigma: 7.8 octaves.

(**f**) Summary data showing z-score values for all recordings in 3 consecutive sessions ('Uncued 1' z-score: 0.4±0.1, 'Self' z-score: 0.1±0.0, 'Uncued 2' z-score: 0.4±0.1, n= 25 recordings, one way ANOVA and Dunnett's multiple comparison test).

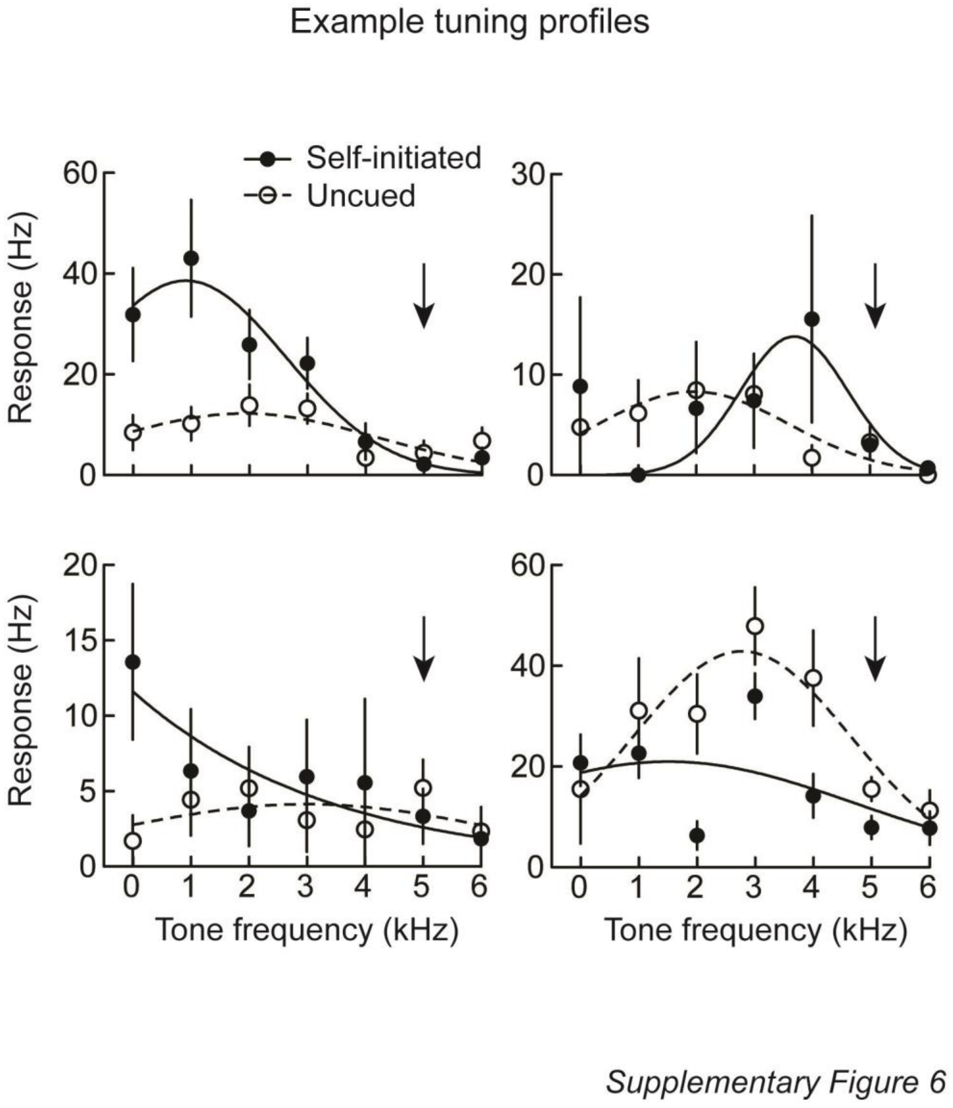

Supplementary Figure 6: Example tuning profiles constructed with firing rates

Representative receptive field modulations by trial self-initiation are shown (two cells from **Supplementary Figure 5** and two additional cells). Gaussian curves were fit to the receptive field distributions for these four cells.

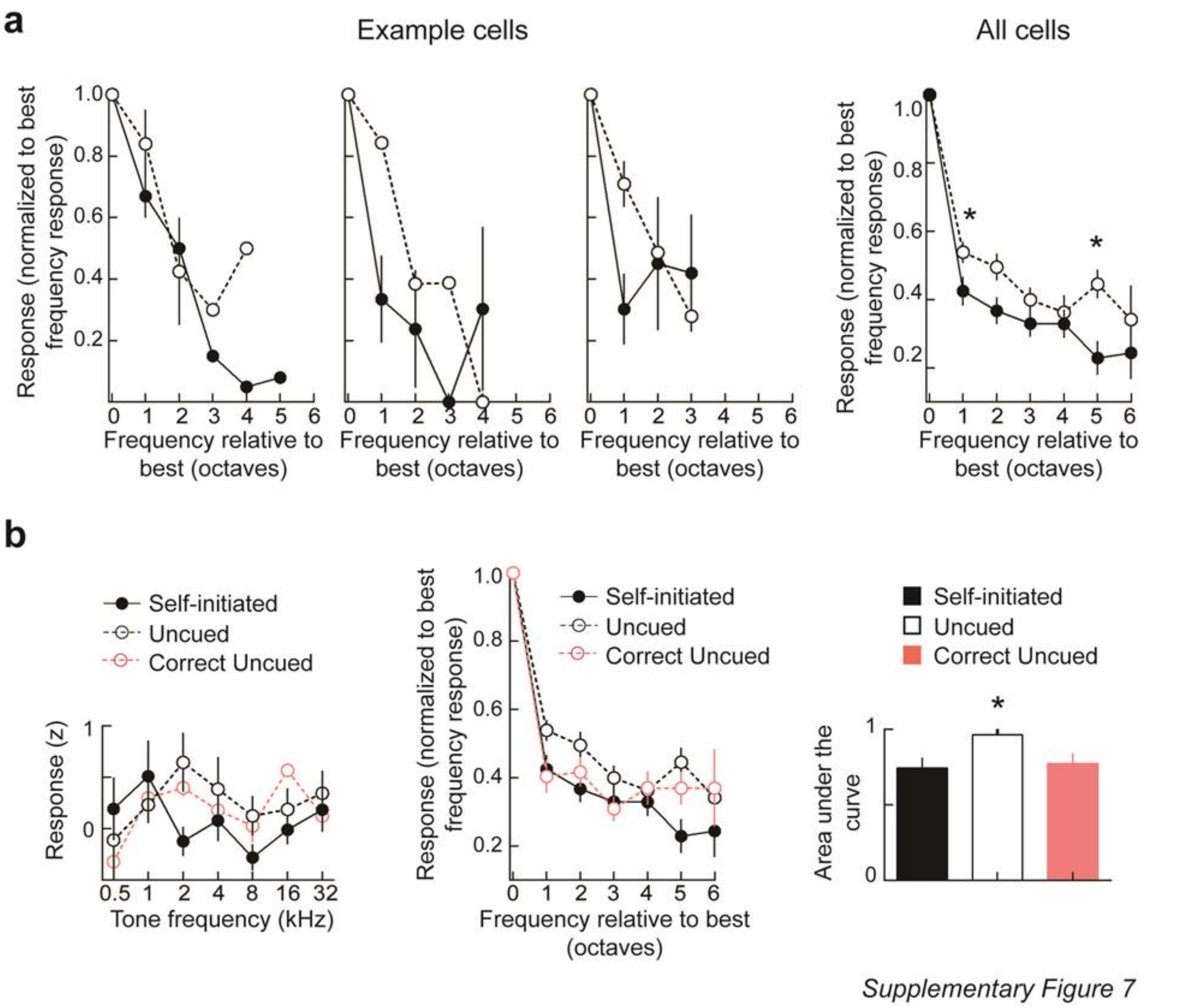

Supplementary Figure 7: Self-initiation sharpens tuning profiles of cortical neurons

(**a**) Left, best frequency-aligned tuning profiles (normalized to the best-frequency response) for the example cells in **Supplementary Figure 5b**. For all cases, self-initiation decreased the area under the curve, which indicates that it sharpened tuning profiles. Right, average of all best

frequency-aligned tuning profiles shows significant decreases in the response to frequencies one and five octaves away from the best frequency of the cell (for the one octave perceptual distance, normalized evoked responses during self-initiated trials were 0.4±0.04 and during uncued trials they were 0.5±0.03, $p<0.03$, ANOVA; for the five octaves perceptual distance, responses were 0.2±0.04 during self-initiated trials and 0.4±0.04 during uncued trials, $p<0.007$).

(**b**) Correct uncued trials (red) have sharper tuning profiles than all uncued trials, similar as the self-initiated trials. Left, example cell tuning profile. Middle, best-frequency aligned tuning plots for self-initiated, uncued and correct uncued trials. Right, area under the curve (AUC) measurements for the aligned tuning profiles (self-initiated AUC was 0.7±0.07, uncued AUC was 0.9±0.05 and correct uncued AUC was 0.7±0.07, $p<0.04$, ANOVA and Dunn's multiple comparison test).

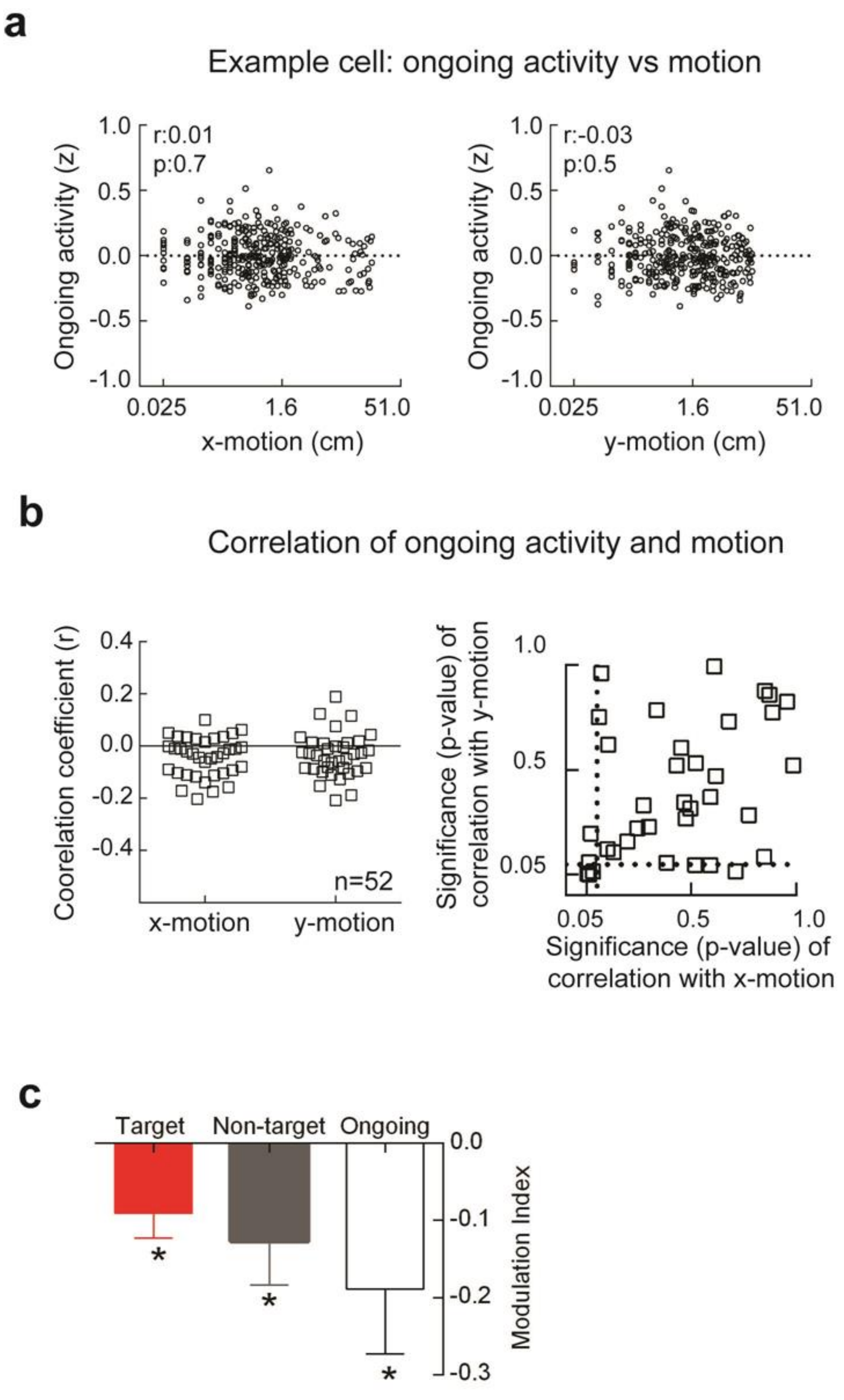

Supplementary Figure 8

Supplementary Figure 8: Ongoing activity in the auditory cortex does not depend on pre-trial movement

(a) Example cell z-score values for ongoing activity as a function of trial-by trial movement during ‘Interval A’ in the x-coordinate (left) and y-coordinate (right).

(b) Spearman correlation coefficients (left) and p-values (right) for all 32 cells recorded. Only 4/32 cells showed significant correlation with either x- or y-motion or both.

(**c**) We selected self-initiated trials that had a similar movement as the uncued trials in each coordinate (±2.5 cm in the x-coordinate and ±5 cm in the y-coordinate) and we calculated a movement-adjusted modulation index between the self-initiated trials and the uncued trials. The modulation index was calculated for target and non-target evoked activity as well as for ongoing activity. We find that even when there was no significant movement preceding the self-initiated trials, neuronal activity was still suppressed by self-initiation as compared to activity during uncued trials (the modulation index was lower than zero: -0.10 ± 0.03, $p<0.02$ for the target tone, -0.13 ± 0.05, $p<0.05$ for non-target tones, and -0.18 ± 0.08, $p<0.05$ for the ongoing activity, one sample t-test).

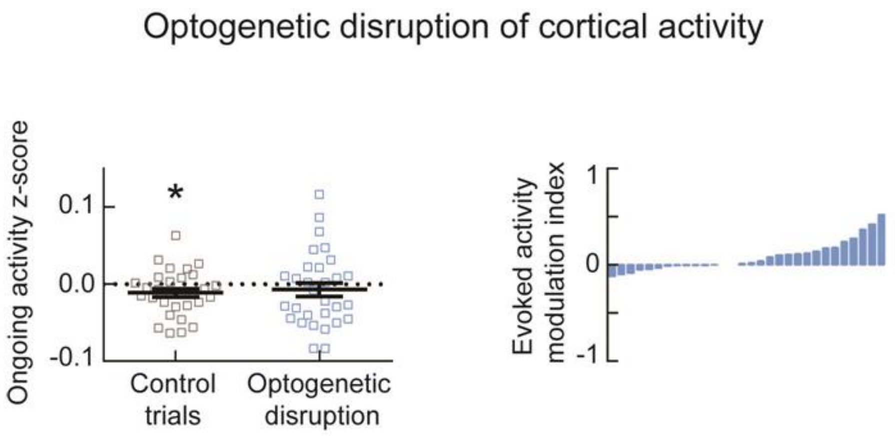

Supplementary Figure 9

Supplementary Figure 9: Optogenetic stimulation disrupts modulation of ongoing activity in the auditory cortex

(a) During control, light OFF, ongoing activity was suppressed by trials self-initiation: mean z-score for the 500 ms preceding tone onset was -0.01±0.00 (n=30 cells, p=0.04, Student's one sample two-tailed t-test). During optogenetically stimulated trials (light ON at self-initiation), ongoing activity was no longer significantly suppressed (mean z-score of ongoing activity was -0.006±0.008, n=30 cells, p=0.4).

(b) Evoked responses are disrupted by optogenetic activation of the auditory cortex. The modulation index between the response during light ON trials and the response during control, light OFF trials was calculated with the formula $(R_{lightON} – R_{lightOFF}) / (R_{lightON} + R_{lightOFF})$. The graph represents the ordered distribution of the modulation index: negative values indicate that light stimulation decreased the evoked response and positive values indicate that light stimulation increased the evoked response. The mean modulation index was 0.08±0.02, and was significantly larger than 0 (p<0.008, n=30, one sample t-test).
